# Research on the impact of residents’ pension insurance choices based on their cognition of pension responsibility

**DOI:** 10.3389/fpubh.2025.1592206

**Published:** 2025-05-07

**Authors:** Lulin Zhou, Wenling Zhu, Yupeng Cui, Yinghua Chen, Xinglong Xu

**Affiliations:** School of Management, Jiangsu University, Zhenjiang, China

**Keywords:** cognition of pension responsibility, multi-pillar pension insurance system, pension willingness, aging, pension insurance choices

## Abstract

**Background:**

The aging of the world’s population has become an increasingly serious issue in recent years, and pensions in all countries have become unsustainable to varying degrees. The sustainability of pension insurance is a pressing issue.

**Objective:**

This study examined Chinese residents’ cognition of pension responsibility and its impact on their participation in pension insurance under the multi-pillar pension insurance system.

**Methods:**

Using data from the 2021 Chinese General Social Survey, the link between residents’ cognition of pension responsibility and pension insurance participation was investigated through binary regression and disordered multiclassified logistic regression analysis.

**Results:**

First, there was a difference in the influence of economic and sociodemographic factors on the choice of basic and commercial pension insurance. Second, “offspring responsibility,” as a dominant perception today, had a negative impact on participation in both basic and commercial pension insurance. The probability of individuals who hold the belief in the “three-party responsibility” participating only in basic pension insurance was significantly higher than that of those who agree with “offspring responsibility” or “self-responsibility.” Third, the relationship between the cognition of pension responsibility and pension insurance participation was moderated by region of residence and people’s access to information.

**Conclusion:**

Through system optimization, awareness campaigns, and education, efforts should be made to modernize and transform the concept of pensions, promote the coordinated development of basic and commercial pension insurance, and build a more comprehensive and sustainable multi-level pension security system.

## 1 Background

The findings of the seventh national population census in 2021 reveal that over 13.5% of China’s population is aged 65 and above, marking an increase of 4.63 percentage points compared to the figures from the sixth national population census in 2010 (1). This indicates that China is on the verge of entering a profoundly aging society. To address the challenges posed by this demographic shift, China has sought inspiration from the theoretical and practical expertise of international organizations in exploring “multi-pillar pension” systems (2). “Developing a multi-level, multi-pillar pension insurance system” was initially introduced in the 2020 Recommendations by the Central Committee of the Communist Party of China on the Formulation of the Fourteenth Five-Year Plan for National Economic and Social Development and the Vision for the 2035 Period (3). As of the present moment, a structural framework for China’s multi-tiered, multi-pillar pension insurance system has been largely put in place.

Nonetheless, Chinese residents have not adequately responded to the government’s encouragement to participate in the multi-pillar pension insurance scheme. According to the China Pension Development Report 2020, published by the Chinese Academy of Social Sciences, by the end of 2019, China’s third pillar of commercial pension insurance had garnered mere hundreds of millions of yuan in premium income, in stark contrast to the approximately 6.3 trillion yuan in accumulated balances within the first pillar of the basic pension insurance fund. A study showed that while an escalating number of residents acknowledge that the burden of older adult care should be shared among multiple entities, the societal belief that children bear primary responsibility remains dominant in society (4). Similarly, the traditional notion of “raising children to support one’s old age” significantly impacts Chinese residents’ willingness to purchase pension insurance (5).

The decisions that residents make regarding pension insurance within a multi-pillar pension insurance system, shaped by their varied perceptions of pension responsibility, hold immense significance for their future well-being in old age, the sustainability of the national pension system, and the future trajectory of China’s pension industry. In light of this “multi-pillar pension insurance system,” do residents have a pension plan in place? Therefore, we examined Chinese residents’ cognition of pension responsibility under the multi-pillar pension insurance system to assess how different cognitions impact their pension insurance choices, with a view to providing a reference for improving the pension policy system and developing the pension service industry.

## 2 Literature review

To explore the various factors influencing residents’ choices in pension insurance, Bateman conducted a survey from the perspective of individual capabilities, asserting that those with financial literacy are more likely to demonstrate an understanding of product characteristics (6). Nonetheless, Rubinstein-Levi revealed that merely boosting financial literacy or utilizing the tax system to encourage more efficient pension savings management has not yielded a substantial impact due to the pension system’s intricacy and dynamic nature (7). Simona employed the “intent-to-decide” analytical framework to model pension choices and the influencing factors. In the segment of the research dedicated to personal pensions, it was concluded that economic factors (such as household economic status and propensity to consume), as well as sociodemographic factors (including gender, education, and marital status), significantly impact pension choices. Based on this, a model was constructed outlining the factors that influence the transition from pension intention to decision (8). In his study on the relationship between gender and pension choices, Foster contended that pension decisions are influenced not solely by gender and age but also by residents’ pension awareness and attitudes. Furthermore, he argued that varying perceptions of pension responsibility impact residents’ planning for their retirement (9). Overburdening of basic pension insurance, intergenerational conflicts, and insufficient financial support affect the willingness of residents to participate in basic pension insurance (10, 11). The concept of pension responsibility in the context of the “raising children for old age” culture significantly inhibits Chinese residents’ willingness to purchase commercial pension insurance (5). It is evident that the selection of pension insurance is influenced not only by economic conditions and sociodemographic factors but also by individuals’ cognitive perceptions. However, there remains ample opportunity for further research to explore how perceptions of pension responsibility impact pension insurance decisions.

Cognition of pension responsibility is one of the components of the concept of pensions. It pertains to individuals’ perspectives, attitudes, and judgments, formed on the basis of objective information and subjective identity, concerning the fundamental issue of who ought to provide pension resources for the older adult (12). Scholars at home and abroad have conducted research around the subject of cognition of pension responsibility, primarily involving the following four kinds of subjects: First is “government responsibility.” Despite fiscal constraints prompting governments to encourage greater personal responsibility among residents, the Western model of older adult care and the cultural milieu emphasizing children’s autonomy have led residents to favor governmental accountability for aging care (13, 14). Second is “offspring responsibility.” Some residents decline to spend their twilight years in nursing homes, adhering to the belief that their offspring ought to shoulder this responsibility (15). Presently, a prevalent influence among many Chinese residents is the cultural norm of “raising children to support oneself in old age,” leading to a preference for “family-based older adult care,” deemed the duty of one’s children (5). The third concept is “self-responsibility” The structural shifts within the multi-pillar pension insurance system necessitate individuals assuming greater responsibility for their own retirement planning. Bernard contended that demographic shifts and fiscal challenges are compelling residents to assume responsibility for their personal pensions and that by emphasizing personal accountability, residents will be incentivized to enroll in voluntary pension schemes (15). Of course, there are researchers who hold that the issue of aging should be addressed collaboratively by the aforementioned three parties, constituting the fourth category, known as “three-party responsibility.” Aart-Jan Riekhoff’s research showed that the sustainability of the pension system depends not only on the ability of the government and society to fund the system but also on the willingness of future generations to support it. Moreover, as trust in the pension system diminishes among the populace, those who possess the financial means will proactively assume responsibility for their own retirement planning (16). The government, children, and individuals should all collaborate to establish a diversified system of older adult care services (17). Therefore, our analysis of cognition of pension responsibility was based on the four viewpoints mentioned above.

The theory of cognitive behavior (TCB) constitutes a comprehensive theory that merges cognitive psychology theories with behaviorist principles, primarily emphasizing the modification of individuals’ thought patterns (18). It suggests that cognitive formations are shaped by ingrained thinking and behavioral patterns. Furthermore, it contends that an individual’s behavior interpretation directly impacts their decision to take action and that changes in both external behavior and internal cognition can affect changes in an individual’s behavior. It is argued that entity responsible for providing pension resources as the bearer of pension responsibilities influences individuals’ participation in pension insurance. Therefore, based on this theory and the relevant literature, we proposed the following hypotheses:

*H1*: The choice of pension insurance is influenced not only by factors such as gender, age, health, education, and financial situation but also by the concept of individuals’ cognition of pension responsibility.

**Figure 1:**
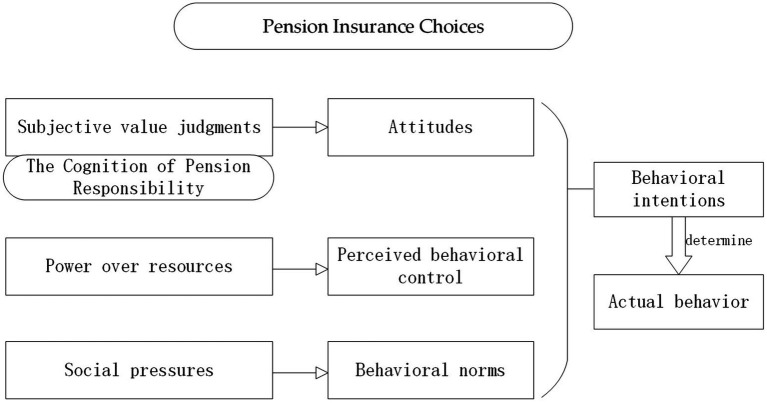
Pension insurance choices.

The theory of planned behavior (TPB) is a social psychological theory used to explain and predict human behavior. The theory suggests that behavioral intentions are the most direct determinants of actual behavior and that behavioral intentions are influenced not only by attitudes (subjective value judgments) and perceived behavioral control (power over resources) but also by behavioral norms (social pressures perceived by the individual) (19). The cognition of pension responsibility corresponds to “attitude,” while health status, education level, and family economic situation correspond to “perceived behavioral control.” Consequently, the influence of behavioral norms on pension insurance participation, such as those shaped by regional culture and social context, must also be taken into account. Thus, a further argumentative hypothesis was established (Figure 1):

*H2*: The relationship between different cognitions of pension responsibility and participation in pension insurance is influenced by behavioral norms.

## 3 Materials and methods

### 3.1 Source of data

The data featured in this article originated from the Chinese General Social Survey 2021 (CGSS2021). CGSS is implemented by Chinese Renmin University in conjunction with universities across the country, adopts stratified multi-stage probability sampling, has strong data representativeness, and is widely used in social science research. Initiated in 2003, it consistently carries out cross-sectional studies encompassing over 10,000 households in mainland China, covering multiple levels of society, community, family, and individuals. A total of 8,148 samples were collected in the 2021 survey, and 7,985 samples were finally included in the analysis (98% effective rate) due to the absence of key variables (such as awareness of pension responsibilities and insurance participation).

### 3.2 Variable values

#### 3.2.1 The explained variable

Given the unique attributes of supplementary pension insurance and its overlap with basic employee pension insurance, this study primarily focused on participation in basic residential pension insurance and commercial pension insurance, using “whether to enroll in pension insurance” as the explanatory variable. Basic pension insurance is a statutory program designed to ensure retirees’ minimum living standards. It comprises two primary schemes: the employee-based plan (for urban workers) and the resident-based plan (covering urban and rural non-workers). Commercial pension insurance, offered by insurers on a voluntary enrollment basis, allows policyholders—individuals or enterprises—to secure retirement income streams through premium payments, with benefits disbursed as stipulated in the policy. It represents a private-sector alternative within the broader pension system.

When basic and commercial pension insurance were the explanatory variables, respectively, “participation” was assigned a value of “1” and “non-participation” a value of “0.” Since the choice of pension insurance under the influence of cognition of pension responsibility is an individual attitude and behavior, it was necessary to comprehensively consider the allocation of individual residents to two types of pension insurance at the same time. As a result, “participation in both” was assigned a value of 1, “basic pension insurance only” a value of 2, “participation in commercial pension insurance only” a value of 3, and “Neither” a value of 4.

#### 3.2.2 The explanatory variables

Based on the question from the CGSS2021 questionnaire, “Who do you think should be responsible for the older adults with children?” and its corresponding answer options—“government responsibility,” “offspring responsibility,” “self-responsibility” and “three-party responsibility”—we can determine the cognition of the main body of responsibility for old age. Specifically, a value of 1 was assigned to “government responsibility,” 2 to “offspring responsibility,” 3 to “self-responsibility” and 4 to “three-party responsibility.”

#### 3.2.3 Control variables

Based on Simona’s “from intention to decision” model of insurance/pension demand and its determinants (8), individual pension decisions are influenced by attitudes (perceived responsibility) (20), ability (economic factors) (21), and context (socio-demographic factors) (22). “Socio-demographic factors” include gender, age, education level, and marital status, among others. And taking into account the reality of China’s urban–rural dual structure, we selected “gender,” “age,” “health status,” “education level,” “family economic situation,” “marital status,” and “nature of household” as control variables for inclusion in our study’s model. Specifically, gender was assigned as “male = 1, female = 2”; age was determined by the respondents’ date of birth according to the United Nations World Health Organization’s definition, and was assigned as “older people = 1, middle-aged people = 2, young people = 3”; health status was assigned as “very unhealthy = 1, relatively unhealthy = 2, average = 3, relatively healthy = 4, very healthy = 5”; education level was assigned as “junior high school and below = 1, senior high school/secondary/technical school = 2, college/undergraduate = 3, graduate and above = 4”; the family economic situation was assigned the values “well below average = 1, below average = 2, average = 3, above average = 4, well above average = 5”; marital status was assigned as “unmarried/cohabiting/divorced/widowed = no spouse = 1, first marriage with spouse/remarriage with spouse/separated but not divorced = spouse = 2”; Hukou nature was assigned as “agricultural hukou = 1, non-agricultural hukou = 2.” The coding of the variables can be found in Table 1.

**Table 1:** Description of variable assignment (*N* = 7,985).

Variant	Value
Basic pension insurance	Yes = 1; no = 0
Commercial pension insurance	Yes = 1; no = 0
Participation in pension insurance	Participation in both = 1; basic pension insurance only = 2; commercial pension insurance only = 3; neither = 4
Cognition of pension responsibility	Government responsibility = 1; offspring responsibility = 2; self-responsibility = 3; three-party responsibility = 4
Gender	Male = 1; female = 2
Age	Older people = 1; middle-aged people = 2; young people = 3
Health status	Very unhealthy = 1; rather unhealthy = 2; average = 3; fairly healthy = 4; very healthy = 5
Education level	Junior high school and below = 1; high school/middle school/technical school = 2; college/undergraduate = 3; postgraduate and above = 4
Family economic situation	Well below average = 1; below average = 2; average = 3; above average = 4; well above average = 5
Marital status	Without spouse = 1; with spouse = 2
Nature of household	Agricultural household = 1; non-agricultural household = 2

#### 3.2.4 Methods of analysis

When analyzing the participation in the two types of pension insurance separately, we employed a Binary Logistic regression model due to the dichotomous nature of the explanatory variable, which categorized participants into “participated” (Yes) and “did not participate (No).”


(1) $$Y_{m}=P_{m}=\alpha_{0}+\overset{n}{\underset{i=1}{\sum }}\alpha_{i}x_{i}+\mu$$

The results of the Binary Regression analysis model can show whether the Cognition of Pension Responsibility has a significant impact on the choice of pension insurance. But when digging deeper to find out exactly which Cognition of Pension Responsibility has an impact on pension insurance choice, it is necessary to rely on the Disordered Multiclassification Regression Model.

In this model we can further learn in what state residents will allocate both basic and commercial pension insurance, in which state they will allocate only basic or commercial pension insurance, and side by side the specific factors that influence when they do not participate in any pension insurance at all. Therefore, when considering individual residents’ choices of two types of pension insurance at the same time, since the explanatory variables “both participate,” “basic only,” “commercial only” and “neither participate” are independent of each other, they are unordered four-category variables (23). And to further analyze exactly how different perceptions of Pension Responsibility affect the choice of pension insurance, we used Disordered Multiclassified Logistic Regression models to analyze the variables (24). Assuming the type of pension insurance participation *Y* = $\left(y_{1},y_{2},\cdots \cdots ,y_{m}\right)$ and the independent variable *X* = ( $x_{1},x_{2},\cdots \cdots ,x_{n}$ ), the model was set up as follows:


(2) $$Y_{m}=\ln\left(\frac{P_{m}}{P_{i}}\right)=\beta_{0}+\overset{n}{\underset{i=0}{\sum }}\beta_{i}x_{i}+\mu$$

In Equations 1, 2, $P_{m}$ is the probability of occurrence of the event $Y_{m}$ (1 ≤ m<i), $P_{i}$ is the probability of occurrence of the reference event $Y_{i}$ , and $P_{1}+P_{2}+\cdots \cdots +P_{m}/P_{i}=1$ ; $P_{m}$ is the probability of the mth group of the explanatory variable, $P_{i}$ is the probability of the ith group of the explanatory variable and the ith group is the reference group; $\alpha_{0}\;\mathrm{and}\;\beta_{0}$ is the constant term; *α* and *β* are the slopes, which are the regression coefficients of the explanatory variables; *μ* is the random error term.

## 4 Results

### 4.1 Distribution of variables

As shown in Table 2, regarding who should be responsible for old age, 55.8% of the total sample endorsed “offspring responsibility,” while only 7.5% believed that the government should be responsible. Among those who endorsed “offspring responsibility,” the lowest proportion participating in basic pension insurance was 68.9%. Additionally, those who agreed that all three parties (children, individuals, and the government) should share responsibility had a higher proportion of participation in commercial pension insurance compared to those holding the other three perceptions of pension responsibility.

**Table 2:** Distribution of variables (*N* = 7,985) (%).

Main variables			Basic pension insurance		Commercial pension insurance		Frequency
			Yes (71.5)	No (28.5)	Yes (7.0)	No (93.0)	
Cognition of pension responsibility	Government responsibility	7.5	76.8	23.2	6.0	94.0	596
	Offspring responsibility	55.8	68.9	31.1	6.1	93.9	4,456
	Self-responsibility	7.9	80.1	19.9	5.7	94.3	632
	Three-party responsibility	28.8	72.7	27.3	9.1	90.9	2,301
Frequency			5,708	2,277	555	7,430	7,985

### 4.2 Model fit results

According to the table below, the results of the chi-square analyses (χ^2^) of the relevant variables (VIF) were significant, and the model did not have the problem of covariance. The impact of residents’ perceptions regarding their pension responsibilities on their decisions to opt for basic or commercial pension insurance within the multi-pillar pension insurance system was significant at the 0.1% level: *χ*^2^ (1, *N* = 7,985) = 46.94, *p* < 0.001, *χ*^2^ (1, *N* = 7,985) = 22.91, *p* < 0.001 (Table 3).

**Table 3:** Model fit results (*N* = 7,985).

Main variables	Basic pension insurance		Commercial pension insurance	
	*χ* ^2^	VIF	*χ* ^2^	VIF
Cognition of pension responsibility	46.937****	1.049	22.906****	1.049
Gender	11.077***	1.015	0.024*	1.015
Age	577.802****	1.438	85.825****	1.438
Health status	77.217****	1.210	37.476****	1.210
Education level	39.938****	1.654	153.510****	1.654
Family economic situation	15.080***	1.125	46.431****	1.125
Marital status	309.432****	1.056	2.413**	1.056
Nature of household	73.605****	1.230	93.638****	1.230

*p < 0.1, **p < 0.05, ***p < 0.01, ****p < 0.001.

### 4.3 Binary regression analysis

The cognition of pension responsibility had a significant effect on the choice of both basic and commercial pension insurance when the control variables were significant, supporting H1.

In the model, some of the variables had different effects on the two dependent variables, basic and commercial pension insurance. In the choice of basic pension insurance, gender, age, and health status had a negative impact on participation in basic pension insurance. In other words, women were less inclined to participate in pension insurance than men. Younger people tended to be less likely to enroll in basic pension insurance. Similarly, healthier individuals were also less inclined to participate in basic pension insurance. In the choice of commercial pension insurance, gender, age, and health status had a positive impact on participation in commercial pension insurance. In other words, women were more likely than men to enroll in commercial pension insurance. Older adults tended to be less inclined to participate in commercial pension insurance. Meanwhile, healthier individuals were more prone to engaging in commercial pension insurance.

Nonetheless, education level, family economic circumstances, marital status, and household registration each exerted a favorable influence on the enrollment in both basic and commercial pension insurance, exhibiting comparable effects. Those who possess higher education qualifications, enjoy a better family economic status, are married, and hold non-agricultural household registrations demonstrated a greater propensity to participate in both types of pension insurance (Table 4).

**Table 4:** Binary regression results (*N* = 7,985).

Main variables	Basic pension insurance		Commercial pension insurance	
	*B*	Std. err.	*B*	Std. err.
Cognition of pension responsibility	0.069**	0.027	0.056*	0.045
Gender	−0.152***	0.053	0.055*	0.091
Age	−0.650****	0.038	0.245****	0.064
Health status	−0.033*	0.027	0.087*	0.049
Education level	0.111***	0.040	0.329****	0.061
Family economic situation	0.088**	0.037	0.169***	0.065
Marital status	0.876****	0.057	0.480****	0.107
Nature of household	0.464****	0.060	0.572****	0.101
Constant term	−0.117*	0.193	−6.378****	0.381
Sample size	7,985		7,985	
Chi-square value	814.70****		219.96****	
-2Log likelihood	−4365.7672		−1905.0944	
Pseudo R^2^	0.085		0.0546	

*p < 0.1, **p < 0.05, ***p < 0.01, ****p < 0.001.

### 4.4 Disordered multiclassified logistic regression analysis

The dependent variable was obtained by taking “neither” as the reference for models 1, 2, and 3. The data for both the independent and dependent variables were obtained based on the last category.

In terms of willingness to participate in both types of insurance, the probability of those who believe in “offspring responsibility” participating in both basic and commercial pension insurance was 75.3% higher than that of those who believe in “three-party responsibility.” Older and middle-aged people were 55.7 and 244.8% more likely than younger people to be enrolled in both basic and commercial pension insurance, respectively. The probability that the very unhealthy group was enrolled in both basic and commercial pension insurance was 43.7% of that of the very healthy group. Residents with junior high school education or less were only 29% as likely as those with graduate school education or higher to be enrolled in both types of pension insurance.

In terms of the results of the willingness to participate only in basic pension insurance, the proportion of those who believe in “offspring responsibility” was only 86.3% of the group that believes in “three-party responsibility.” The proportion of those who believe in “self-responsibility” was 95.6% of those who believe in “three-party responsibility,” with almost no difference. The proportion of men with only basic pension insurance was 14.9% higher than that of women with only basic pension insurance. Older and middle-aged people were 311.6 and 235.8% more likely than young people to be covered only by basic pension insurance. The probability of being enrolled only in basic pension insurance was 18.8 and 17.1% higher for the generally healthy and relatively healthy groups, respectively, than for the very healthy group.

In terms of the results of the willingness to participate only in commercial pension insurance, the probability that those who believe in “offspring responsibility” participating only in commercial pension insurance was 78.9% higher than that of those who believe in “three-party responsibility.” Middle-aged people were 175.9% more likely than young people to be enrolled only in commercial pension insurance. The probability of the relatively unhealthy group being only enrolled in commercial pension insurance was only 44.1% of the probability of the very healthy group.

At the same time, the probability of being enrolled in both or only one type of pension insurance was significantly higher for those in a better family economic situation, those with spouses, and the non-agricultural household group than for those in a worse family economic situation, those without spouses, and the agricultural household group (Table 5).

**Table 5:** Disordered multiclassified logistic regression results (*N* = 7,985).

Variables		Participation in both/neither (Model 1)		Basic only/neither (Model 2)		Commercial only/neither (Model 3)	
		B	Exp(B)	B	Exp(B)	B	Exp(B)
Cognition of pension responsibility (reference groups = 4)	Government responsibility	−0.236	0.790	−0.131	0.877	0.225	1.253
		(0.239)		(0.119)		(0.404)	
	Offspring Responsibility	−0.284**	0.753	−0.147**	0.863	−0.237**	0.789
		(0.119)		(0.064)		(0.245)	
	Self-responsibility	−0.236	0.790	−0.045*	0.956	−0.065	0.937
		(0.236)		(0.121)		(0.443)	
Gender (reference groups = 2)	Male	0.116	1.123	0.139**	1.149	−0.306	0.737
		(0.107)		(0.055)		(0.221)	
Age (reference groups = 3)	Older people	0.443***	1.557	1.415****	4.116	0.568*	1.764
		(0.166)		(0.078)		(0.317)	
	Middle-aged people	1.238****	3.448	1.211****	3.358	1.015****	2.759
		(0.142)		(0.080)		(0.289)	
Health status (reference groups = 5)	Very unhealthy	−0.829*	0.437	0.094	1.098	−0.524	0.592
		(0.448)		(0.141)		(0.652)	
	Rather unhealthy	0.148	0.862	0.063	1.065	−0.819*	0.441
		(0.224)		(0.104)		(0.488)	
	Average	0.080	1.084	0.172**	1.188	−0.071	0.931
		(0.157)		(0.083)		(0.310)	
	Fairly healthy	0.082	1.086	0.158**	1.171	0.025	1.025
		(0.141)		(0.076)		(0.281)	
Education level (reference groups = 4)	Junior high school and below	−1.239****	0.290	−0.212	0.809	−0.930	0.395
		(0.345)		(0.227)		(0.605)	
	High school/middle school/technical school	−0.534	0.586	−0.156	0.855	−0.845	0.430
		(0.337)		(0.227)		(0.599)	
	College/undergraduate	−0.112	0.894	0.058	1.059	−0.918	0.399
		(0.323)		(0.222)		(0.580)	
Family economic situation (reference groups = 5)	Well below average	−2.745***	0.064	−1.506**	0.222	13.626****	827015.422
		(0.873)		(0.746)		(0.542)	
	Below average	−2.547***	0.078	−1.483**	0.227	13.854****	1038854.109
		(0.844)		(0.741)		(0.377)	
	Average	−2.350***	0.095	−1.390*	0.249	13.707****	897580.138
		(0.840)		(0.741)		(0.354)	
	Above average	−1.906**	0.149	−1.284*	0.277	14.295	1614673.445
		(0.852)		(0.747)		(0.000)	
Marital status (reference groups = 2)	Without spouse	−0.940****	0.390	−0.796****	0.451	−0.482**	0.617
		(0.127)		(0.060)		(0.391)	
Nature of household (reference groups = 2)	Agricultural household	−0.849****	0.428	−0.442****	0.642	−0.714***	0.489
		(0.122)		(0.063)		(0.388)	
Constant term		0.981**		2.060***		−15.542****	
		(0.902)		(0.773)		(0.654)	
Sample size		7,985					
Chi-square value		1222.906****					
-2Log likelihood		5902.021					
Pseudo R^2^		0.090					

*p < 0.1, **p < 0.05, ***p < 0.01, ****p < 0.001.

### 4.5 Robustness check

Robustness tests were conducted using the model replacement method. The original disordered multiclassified logistic regression model used was replaced with a probit model, based on the fact that both models examined the intrinsic connection between the outputs and inputs of interest by probabilistically modeling the response variables and estimating these model parameters using the usual maximum likelihood parameter fitting approach. According to Table 6, the significance of the variables and the direction of the regression coefficients were the same for both models, and the models were stable. Notably, despite the reduction in data size from previous surveys in the CGSS2021 dataset, the results of the study show that these data are still of research significance and universal value (Table 7).

**Table 6:** Probit robustness tests for the binary regression analysis.

Variables	Basic		Commercial	
	Logistic	Probit	Logistic	Probit
Cognition of pension responsibility	0.069**	0.041**	0.056*	0.028*
	(0.027)	(0.016)	(0.045)	(0.022)
Gender	−0.152***	−0.084***	0.055*	0.028*
	(0.053)	(0.031)	(0.091)	(0.045)
Age	−0.650****	−0.377****	0.245****	0.121****
	(0.038)	(0.022)	(0.064)	(0.031)
Health status	−0.033*	−0.018*	0.087*	0.040*
	(0.027)	(0.016)	(0.049)	(0.024)
Education level	0.111***	0.061***	0.329****	0.165****
	(0.040)	(0.024)	(0.061)	(0.031)
Family economic situation	0.088**	0.051**	0.169***	0.083***
	(0.037)	(0.022)	(0.065)	(0.031)
Marital status	0.876****	0.517****	0.480****	0.225****
	(0.057)	(0.034)	(0.107)	(0.052)
Nature of household	0.464****	0.280****	0.572****	0.281****
	(0.060)	(0.035)	(0.101)	(0.049)
Constant term	−0.117*	−0.089*	−6.378****	−3.305****
	(0.193)	(0.115)	(0.381)	(0.182)

**Table 7:** Probit robustness tests for the disordered multiclassified logistic regression analysis.

Variables	Logistic			Probit		
	Model 1	Model 2	Model 3	Model 1	Model 2	Model 3
Cognition of pension responsibility	−0.236	−0.131	0.225	−0.123	−0.087	0.052
	(0.239)	(0.119)	(0.404)	(0.122)	(0.080)	(0.171)
	−0.284**	−0.147**	−0.237**	−0.126**	−0.082*	−0.101
	(0.119)	(0.064)	(0.245)	(0.197)	(0.044)	(0.100)
	−0.236	−0.045*	−0.065	−0.127	−0.028	−0.034
	(0.236)	(0.121)	(0.443)	(0.121)	(0.079)	(0.181)
Gender	0.116	0.139**	−0.306	0.060	0.076**	−0.088
	(0.107)	(0.055)	(0.221)	(0.056)	(0.037)	(0.089)
Age	0.443***	1.415****	0.568*	0.148***	0.395****	0.172***
	(0.166)	(0.078)	(0.317)	(0.041)	(0.027)	(0.062)
Health status	−0.829*	0.094	−0.524	−0.023	0.017	−0.052
	(0.448)	(0.141)	(0.652)	(0.030)	(0.019)	(0.045)
Education level	−1.239****	−0.212	−0.930	−0.202****	−0.032	−0.033
	(0.345)	(0.227)	(0.605)	(0.040)	(0.029)	(0.063)
Family economic situation	−2.745***	−1.506**	13.626****	−0.122***	−0.042	0.040
	(0.873)	(0.746)	(0.542)	(0.040)	(0.026)	(0.060)
Marital status	−0.940****	−0.796****	−0.482**	−0.554****	−0.508****	−0.322***
	(0.127)	(0.060)	(0.391)	(0.065)	(0.041)	(0.097)
Nature of household	−0.849****	−0.442****	−0.714***	−0.430****	−0.262****	−0.327***
	(0.122)	(0.063)	(0.388)	(0.062)	(0.042)	(0.096)
Constant term	0.981**	2.060***	−15.542****	2.567***	0.163	−2.368****
	(0.902)	(0.773)	(0.654)	(0.231)	(0.141)	(0.350)

*p < 0.1, **p < 0.05, ***p < 0.01, ****p < 0.001.

### 4.6 Endogeneity test

The participation of our residents in pension insurance is influenced by variables such as the perception of pension responsibility, but this is not the result of random assignment. In order to effectively minimize the impact of errors due to selection errors, endogenous type tests were performed using propensity score matching (PSM). K Near-neighbor matching, radius matching, and kernel matching methods were used for the analysis to avoid the bidirectional causal effect of perceptions of pension responsibility on the pension insurance participation of our residents (25). A self-sampling method (Bootstrap) was utilized to estimate the standard error of the statistic and implement the statistical inference.

The results show that the values of ATT for K-nearest neighbor matching, radius matching, and kernel matching are significant. After controlling for selection bias, perceptions of old-age responsibility have a significant effect on the participation of our residents in old-age insurance (Table 8).

**Table 8:** The ATT effect of cognition of pension responsibility on pension insurance participation.

Variable	Matching methods	ATT	S.E.	*t*
Y	K-nearest neighbor matching	2.52***	0.022	4.07
	Radius matching	2.56*	0.021	1.88
	Kernel matching	2.56*	0.023	1.86

*p < 0.1, **p < 0.05, ***p < 0.01, ****p < 0.001.

### 4.7 Heterogeneity analysis

To further analyze the tendency of young and middle-aged people to choose pension insurance participation under different cognitions of pension responsibility, based on the United Nations World Health Organization’s definition of age, two different groups of young people and middle-aged and older people were established using the age of 44 as the dividing line.

Among young people, a lower level of education was found to have a notable negative effect on their pension insurance choices. However, this effect was not significant among middle-aged and older adults. Among middle-aged and older adult people, the belief “self-responsibility” had a negative influence on the behavioral choice of pension insurance, which was significant at the 10% level. Additionally, the family’s financial status significantly influenced the behavioral choice of purchasing commercial pension insurance, as shown in Table 9.

**Table 9:** Heterogeneity analysis of the impact of the cognition of pension responsibility on pension insurance participation.

Variables		Young people			Middle-aged and older people		
		Model 1	Model 2	Model 3	Model 1	Model 2	Model 3
Cognition of pension responsibility (reference groups = 4)	Government responsibility	−0.580	−0.097	0.931	−0.343	−0.171	−0.144
		(0.642)	(0.311)	(0.807)	(0.267)	(0.135)	(0.463)
	Offspring Responsibility	−0.367**	−0.116	−0.063	−0.160	−0.197**	−0.366
		(0.172)	(0.097)	(0.388)	(0.169)	(0.090)	(0.319)
	Self-responsibility	0.323	0.489	0.658	−0.481*	−0.163	−0.418
		(0.547)	(0.327)	(1.085)	(0.268)	(0.135)	(0.488)
Gender (reference groups = 2)	Male	0.635***	0.431***	0.391	0.244*	−0.035	−0.734**
		(0.165)	(0.092)	(0.357)	(0.144)	(0.071)	(0.283)
Health status (reference groups = 5)	Very unhealthy	−16.790	1.166**	−17.629	−1.256***	−0.067	−0.821
		(5829.768)	(0.472)	(0.000)	(0.466)	(0.162)	(0.681)
	Rather unhealthy	0.280	0.094	−17.968	−0.613**	−0.038	−1.041**
		(0.465)	(0.267)	(7035.344)	(0.272)	(0.130)	(0.527)
	Average	0.235	0.327	0.352	−0.310	−0.022	0.579
		(0.238)	(0.127)	(0.480)	(0.220)	(0.120)	(0.403)
	Fairly healthy	0.144	0.123	0.156	−0.159	0.086	−0.243
		(0.190)	(0.103)	(0.412)	(0.216)	(0.121)	(0.388)
Education level (reference groups = 4)	Junior high school and below	−1.673***	−0.750***	−1.003	−1.584*	0.374	−1.566
		(0.434)	(0.255)	(0.751)	(0.885)	(0.788)	(1.270)
	High school/middle school/technical school	−0.947**	−0.656***	−1.630**	−0.703	0.586	−0.993
		(0.405)	(0.250)	(0.783)	(0.885)	(0.791)	(1.274)
	College/undergraduate	−0.146	−0.150	−0.917	−0.314	0.594	−1.372
		(0.370)	(0.237)	(0.669)	(0.893)	(0.800)	(1.324)
Family economic situation (reference groups = 5)	Well below average	−0.580**	−0.035	−0.304	−2.756***	−1.288*	14.617****
		(0.294)	(0.178)	(0.566)	(0.891)	(0.745)	(0.692)
	Below average	−0.281	−0.053	−0.778	−2.323***	−1.194	15.028****
		(0.254)	(0.165)	(0.533)	(0.850)	(0.740)	(0.505)
	Average	−0.580**	−0.035	−0.304	−2.225***	−1.015	15.067****
		(0.294)	(0.178)	(0.566)	(0.846)	(0.740)	(0.483)
	Above average	−0.281	−0.053	−0.778	−1.686*	−0.798	15.500
		(0.254)	(0.165)	(0.533)	(0.870)	(0.755)	(0.000)
Marital status (reference groups = 2)	Without spouse	−1.722***	−1.493***	−1.046***	−0.264	−0.239***	0.021
		(0.180)	(0.101)	(0.391)	(0.180)	(0.084)	(0.313)
Nature of household (reference groups = 2)	Agricultural household	−0.539***	−0.296***	−0.449	−0.987****	−0.532****	−0.800**
		(0.178)	(0.097)	(0.388)	(0.172)	(0.088)	(0.310)
Constant term		0.114	1.122***	−1.435**	3.348***	2.583**	−14.950****
		(0.456)	(0.291)	(0.857)	(1.228)	(1.084)	(1.327)

*p < 0.1, **p < 0.05, ***p < 0.01, ****p < 0.001.

### 4.8 Moderating effects analysis

To further explore the influence of behavioral norms (regional culture and social context) on the relationship between pension responsibility and pension participation, according to the theory of planned behavior, we added two moderating variables—region of residence and commonly used social media—for a deeper analysis of the model (28). Since perceptions of pension responsibility are categorical variables, moderating effects were analyzed using hierarchical regression after centering the variables.

The ∆F significance results indicate that the region in which residents live and their daily access to information moderated the relationship between pension responsibility and beliefs about pension participation. Specifically, the region of residence had a negative moderating effect on the participation of those who belief “offspring responsibility” and a positive moderating effect on those who belief “self-responsibility.” The source of information negatively moderated participation in pension insurance among those who belief “government responsibility.” Therefore, H2 is supported (Tables 10, 11).

**Table 10:** Analysis of the moderating variables for districts.

Variables	Model 1	Model 2	Model 3
	B	B	B
Constant term			
X1	−0.010	−0.010	0.017
X2	0.059****	0.058****	0.144****
X3	−0.030**	−0.031**	0.036
Districts		0.016	0.076****
Districts-X1			−0.031
Districts-X2			−0.111***
Districts-X3			0.078***
R^2^	0.006	0.006	0.008
Adjusted R^2^	0.006	0.006	0.007
∆R^2^	0.006	0.000	0.002
∆F	14.524****	1.944	4.219***
Y: Participation in pension insurance			

*p < 0.1, **p < 0.05, ***p < 0.01, ****p < 0.001.

**Table 11:** Analysis of the moderating variables for information sources.

Variables	Model 1	Model 2	Model 3
	B	B	B
Constant term			
X1	−0.010	−0.002	0.140*
X2	0.059****	0.062****	−0.013
X3	−0.030**	−0.023*	0.076
Information sources		0.055****	0.058**
Information sources-X1			−0.144*
Information sources-X2			0.077
Information sources-X3			−0.100
R^2^	0.006	0.009	0.010
Adjusted R^2^	0.006	0.008	0.009
∆R^2^	0.006	0.003	0.001
∆F	14.524****	21.676****	3.085**
Y: Participation in pension insurance			

*p < 0.1, **p < 0.05, ***p < 0.01, ****p < 0.001.

## 5 Discussion

The current aging problem is gradually worsening, and despite the enormous pension pressure, the inauguration of China’s multi-pillar pension insurance system has not received a positive response from residents. Among these, the basic pension insurance system, which is the “only one” under China’s pension insurance system, has undergone nearly 70 years of development and evolution and has always assumed the function of guaranteeing the basics. The “second pillar” of enterprise annuities and occupational annuities, also known as supplementary pension insurance, has faced the issue of insufficient coverage since its implementation. Only a very small number of enterprises and units provide insurance coverage for laborers on top of the basic endowment insurance. Commercial pension insurance as the “third pillar” can, to a certain extent, supplement the shortcomings of basic pension insurance and enterprise supplementary pension insurance. However, the development of commercial pension insurance started later than basic pension insurance, and its enrollment rate for residents is low.

The results showed that economic and sociodemographic factors significantly affect the willingness of residents to participate in pension insurance. In addition to this, the “consciousness-to-decision” model and the theory of cognitive behavior (TCB) affirm the positive effect of an individual’s subjective awareness on the behavior itself. Therefore, we proposed H1. Based on the behavioral norms factor of the theory of planned behavior (TPB), we formulated H2.

### 5.1 Differential impact of economic and sociodemographic factors on the choice of basic and commercial pension insurance

#### 5.1.1 Demographic factors affect the willingness to participate in pension insurance differently

Gender, age, and health status have a negative effect on participation in basic pension insurance and a positive effect on participation in commercial pension insurance.

In terms of gender, women are less likely than men to participate in basic pension insurance, which may be related to labor market participation and the division of social roles. Women tend to face more instability in the job market, such as career breaks and informal employment, which may lead to a disadvantage in their participation in basic pension insurance. However, the fact that women tend to participate in commercial pension insurance more than men may be related to them being more risk-conscious and capable of long-term planning. Additionally, women’s preference for commercial pension insurance can help make up for the shortfall in their participation rate in basic pension insurance and improve their overall level of pension security.

In terms of age, younger people are less inclined to participate in basic pension insurance, mainly because they have a lower sense of urgency with regard to pension issues. In addition, some young people may lack understanding of the system and benefits of basic pension insurance, which further reduces the incentive to participate in the scheme. The older the persons, the less likely they are to participate in commercial pension insurance, mainly because commercial pension insurance usually has a certain limit on the age of participation, and the older the age, the higher the premiums and the lower the returns.

In terms of health status, healthy people are less likely to participate in basic pension insurance, probably because they perceive less future pension risks (e.g., medical expenses and risk of incapacitation). This may expose healthy populations to the risk of inadequate coverage in the event of future changes in their health status, especially as medical and long-term care needs increase. However, healthy people are more inclined to enroll in commercial pension insurance, probably because they are more likely to pass the insurance company’s health underwriting, thus obtaining more favorable premiums and more comprehensive coverage. In addition, healthy people have more optimistic expectations for their future lives and are more willing to secure additional protection for their old age through commercial insurance. Healthy people can obtain more favorable protection conditions through commercial pension insurance, which not only enhances their level of pension protection but also provides additional support for future health risks.

Taken together, the differential impact of basic and commercial pension insurance in terms of gender, age, and health status reflects the different positioning and functions of the two insurance systems. Basic pension insurance, as a basic system, is more influenced by the compulsory nature of the system and social roles, while commercial pension insurance, as a complementary system, is more influenced by individual risk awareness, financial ability, and health status.

#### 5.1.2 Economic and social factors have the same effect on the willingness to participate in both types of pension schemes

People who are highly educated tend to participate in pension insurance, perhaps because they are more cognizant and risk-averse and usually have a clearer understanding of pension risks. As a result, they are better able to understand the importance of pension insurance and the mechanisms by which it operates, and they are more inclined to diversify pension risks through institutionalized means (e.g., basic and commercial pension insurance). Their participation in both basic and commercial pension insurance helps build a multi-level pension insurance system. At the same time, their needs and expectations for pension insurance may drive the innovation of insurance products and the improvement of the system.

People with a better family economic situation has a stronger financial ability to pay for pension insurance premiums, especially commercial pension insurance premiums, which provides a material basis for their participation in the insurance scheme. Moreover, they may have higher expectations for their pension life and prefer to supplement basic pension insurance through commercial pension insurance. They are able to choose higher-grade contribution rates and more comprehensive insurance products, thus enhancing the level of retirement protection. The choice of commercial pension insurance by groups with better family economic situations provides an impetus for the development of the insurance market and helps to promote commercial insurance development.

People with spouses usually have a stronger sense of responsibility for the family and are more inclined to provide for their own and their spouse’s old age. Married couples tend to plan for the future, including retirement, together, and this sense of joint planning leads to more active participation in pension insurance. Their participation behavior not only protects themselves, but married couples may be more inclined to participate in pension insurance together when they participate, which tends to create a stronger synergy of protection.

With regard to the type of hukou, non-agricultural hukou groups are more likely to be covered by the basic pension insurance system, especially in the urban workers’ pension insurance system, where they have more opportunities to participate. Moreover, non-agricultural household groups usually enjoy more economic and social resources and are better able to afford commercial pension insurance. The high participation rate in basic pension insurance and preference for commercial pension insurance among this group not only help to expand the coverage of basic pension insurance but also provide room for the expansion of the insurance market.

Taken together, education level, family economic situation, marital status, and hukou type have a positive impact on participation in both basic and commercial pension insurance, reflecting the important role of socioeconomic factors and personal characteristics in the choice of pension insurance. These factors not only affect residents’ willingness and ability to participate but also shape the overall level of their old-age security.

### 5.2 Cognition of pension responsibility significantly influences residents’ choice of pension insurance

#### 5.2.1 Offspring responsibility

The perception of “offspring responsibility” is still prevalent today and has a negative impact on participation in basic and commercial pension insurance. This finding is generally consistent with the findings of the study mentioned above, indicating that “raising children for old age” inhibits residents’ willingness to participate in pension insurance.

From the perspective of traditional cultural concepts, the majority of Chinese residents are influenced by the cultural atmosphere of “raising children for old age” and prefer “offspring responsibility” (5). Although there is a growing trend of urban older persons choosing to rely on themselves for their old age, there has been no fundamental change in the traditional notion of rural older persons of raising children for their old age. This perception undermines their willingness to solve their old-age problems through institutionalized means (e.g., pension insurance).

In terms of the specificity of the family structure, in traditional Chinese culture, the family is the core unit for old age, and intergenerational support between parents and children is seen as a moral obligation. This family model of old age has led to some residents feeling less of a need for pension insurance, believing that the financial support and care of their children is sufficient to meet their old-age needs (5). But this traditional family structure is changing, and this change has had a somewhat dismantling effect on the perception that children are responsible for old age, weakening the sustainability of the traditional model. First, the miniaturization of family size has contributed to the separation of generations. The popularization of mechanized agricultural production in rural areas has reduced the dependence on young and middle-aged laborers, and the agricultural inheritance model of “sons inheriting from their fathers” has been broken (26). The urban migration of the younger generation has led to an increase in the proportion of “empty nesters,” and physical isolation has reduced the feasibility of daily care. Second, declining fertility rates have led to a break in the intergenerational chain of support. Most families will face a “4–2-1” structure (4 grandparents, 2 parents, 1 child), making it difficult for a single offspring to take on multiple support responsibilities. Finally, the transformation of women’s roles has led to a shrinking of care resources. The breakdown of the traditional gender division of labor and a female labor force participation rate of 61% (higher than the OECD average) have weakened the supply of unpaid care within the family (27).

However, the inhibiting effect of the perception of “offspring responsibility” still has impacts on pension insurance purchasing behavior. First, the willingness to participate in insurance is lowered. Residents with the perception of “offspring responsibility” tend to believe that pension insurance is superfluous and, therefore, have a lower willingness to participate in basic and commercial pension insurance. Second, it weakens risk awareness. The perception of dependence on children for old age may lead residents to ignore the risks of old age. They may underestimate the financial pressures, medical costs, and uncertainty about their children’s ability to support them in the future as they age, thus reducing the need for pension insurance. Third, it affects the sustainability of contributions. Even if some residents are enrolled in pension insurance, those with the perception of “offspring responsibility” may attach less importance to pension insurance and may be more likely to discontinue contributions or choose a lower level of contribution when faced with financial pressures. Fourth, it impedes the construction of a multilevel old-age security system. The concept of over-reliance on children may lead to insufficient participation by residents in basic and commercial pension insurance, thus affecting the popularization and improvement of the multi-level pension insurance system and impeding the enhancement of the level of personal pension security.

#### 5.2.2 Three-party responsibility

From the perspective of only participating in basic pension insurance, the probability of participating in pension insurance is significantly higher among those who believe in “three-party responsibility” than among those who believe in “offspring responsibility” and “self-responsibility.”

This conclusion is perhaps due to the modernity and comprehensiveness of the perception of “three-party responsibility.” First, the concept emphasizes the shared responsibility of individuals, families, and society for old age. It is more in line with the direction of development of the modern social security system. Residents with this perception are more inclined to institutionalize their old age rather than relying solely on their children or personal savings. Second, the perception emphasizes the awareness of risk diversification. This perception reflects a comprehensive understanding of pension risks, as residents realize that it is difficult to rely solely on their children or themselves to cope with possible future pension risks (e.g., longevity risks and rising medical costs) and are, therefore, more willing to diversify risks through basic pension insurance. Third, this perception strengthens individuals’ trust in socialized security. The perception of “three-party responsibility” reflects residents’ trust in and reliance on the socialized old-age security system, believing that the country and society should take important responsibility for old-age security.

This perceived high probability of pension insurance participation, based on the “three-party responsibility,” is viewed from the perspective of its impact on pension insurance purchasing behavior. First, it helps to increase the willingness to participate. Residents with the perception of “three-party responsibility” are more likely to participate in basic pension insurance. This is because they recognize that individual contributions are only part of the responsibility for old age and that the government and society also bear an important responsibility. Second, it helps to promote institutional identity. The perception of “three-party responsibility” helps to strengthen residents’ sense of identity and trust in the basic pension insurance system, thus promoting the popularization and optimization of the system. Third, it helps to make up for the inadequacy of family and individual retirement. By participating in basic pension insurance, residents with the perception of “three-party responsibility” can effectively make up for the inadequacy of family pension and personal savings and provide more comprehensive protection for their future retirement life. Fourth, it helps to promote the construction of a multi-level security system. This perception not only promotes participation in basic pension insurance but also lays the foundation for residents’ future participation in supplementary protection, such as commercial pension insurance, which helps build a multi-level pension insurance system.

#### 5.2.3 Self-responsibility

The difference between the perceptions of “self-responsibility” and “three-party responsibility” for pension insurance is very small in terms of the impact of participation in basic pension insurance only. This may be analyzed for the following reasons:

First, basic pension insurance itself is compulsory and universal. Whether residents have the perception of “self-responsibility” or “three-party responsibility,” as long as they are covered by the system, they will participate in basic pension insurance either passively or actively. This institutional design undermines differences in the impact of different cognitions of pension responsibility on enrollment behavior. Second, cognitive differences are weakened at the level of basic security. At the level of basic pension insurance, differences in residents’ cognition of pension responsibility sharing may be downplayed. Since basic pension insurance reflects more of the State’s and society’s commitment to residents’ pension responsibility, individual contributions are only a part of it. Third, the impact of policy advocacy and institutional popularization. The government has made greater efforts to publicize and promote basic pension insurance, and there is a high degree of consensus among residents on its importance and necessity. This consensus goes some way to bridging the behavioral differences between the different cognition of pension responsibility groups.

This phenomenon may have the following implications for pension insurance purchasing behavior: First, the convergence of enrollment behavior. Residents with both “self-responsibility” and “three-party responsibility” perceptions show a high degree of consistency in their behavioral choice to participate only in basic pension insurance. This shows that basic pension insurance, as a basic system, is able to cover the pension security needs of different cognizant groups. Second, potential differences in contribution brackets. Despite smaller differences in enrollment behavior, the two cognitive groups may differ in their choice of contribution bracket. For example, residents with the perception of “self-responsibility” may be more inclined to choose a higher level of contribution to enhance their personal retirement protection, while residents with the perception of “three-party responsibility” may pay more attention to balancing the sharing of responsibilities among the individual, the family, and society. Third, different attitudes toward institutional dependence. Residents with the perception of “self-responsibility” are likely to focus more on personal savings and investment, while those with the perception of “three-party responsibility” are likely to rely more on institutionalized protection systems. This difference in attitudes may be even more pronounced in pension planning beyond basic pension insurance.

It is worth noting that although the difference in the impact of “self-responsibility” and “three-party responsibility” on the behavioral choices to only participate in basic pension insurance is relatively small, this difference may change as the pension insurance system is reformed and improved. For example, the behavioral choices of different cognition groups may further diverge if the contribution and entitlement mechanisms of basic pension insurance become more flexible and diverse. In addition, as the income level of residents increases and the concept of pension changes, the difference in the impact of the two perceptions on enrollment behavior may become more evident in other areas of pension security (e.g., commercial pension insurance and personal pension accounts).

### 5.3 The cognition of pension responsibility and pension insurance choice behavior among the group of young people

Influenced by social issues such as population aging and increasing pressure on social security, younger generations (especially the Post-90s and Post-00s) are generally cognizant of the need to self-fund their retirement. However, they lack a clear path for specific planning. On the one hand, the “procrastination mindset” leads young people to view aging as a “distant concern,” thereby directing their focus toward immediate financial obligations such as purchasing a home and funding education, which take precedence in their short-term planning. On the other hand, certain young individuals aspire to substitute conventional old-age insurance with alternatives like “senior housing” or financial instruments (such as mutual funds and equities).

In terms of participation in basic old-age insurance, the participation behavior of young people is characterized by significant passivity due to the mandatory withholding of contributions by enterprises in accordance with the law, and the overall participation rate is maintained at a high level. However, the group’s knowledge of the rules of the pension system (e.g., core parameters such as the minimum number of years of contribution, pension replacement rate, etc.) is generally insufficient, resulting in a pronounced issue of information asymmetry. In addition, surveys of the flexible employment group show that their participation behavior is significantly influenced by the sensitivity of the contribution burden. Flexible workers are subject to a higher percentage of contribution obligations (20% of the contribution base under the current policy) than those in formal employment, which further undermines their confidence in the level of future pension benefits they expect to receive (28).

In terms of participation in commercial pension insurance, the willingness of younger groups to participate in the commercial pension insurance sector is differentially driven. On the one hand, tax incentives (e.g., the tax-deferred effect of tax-deferred pension insurance) and product flexibility (e.g., the premium add-on and partial withdrawal features of universal life insurance) are significantly attractive to high-income, financially literate subgroups. On the other hand, participation behavior still faces obstacles of budget constraints and institutional trust. For example, there is resource competition between premium spending and short-term consumption needs (e.g., housing rental, leisure and entertainment), and some respondents are skeptical about the long-term solvency and contract enforcement rigidity of insurance companies (29).

### 5.4 The relationship between the cognition of pension responsibility and pension insurance participation is moderated by region and access to information

According to the theory of planned behavior, behavioral norms are the social pressures or expectations that an individual feels when deciding whether or not to adopt a behavior. It is determined by a combination of normative beliefs and compliance motives that reflect an individual’s perception of social expectations and willingness to comply. From a behavioral norms perspective, region and information access moderate the relationship between beliefs about pension responsibility and pension insurance participation behavior by influencing residents’ normative beliefs and compliance motives.

Specifically, residents in the western region tend to strongly agree that their children should be responsible for their old age, whereas residents in the eastern region are more inclined to believe that they should be personally responsible for their own care in later life. This may be because in the western region, where traditional attitudes are strong and residents generally believe that their children should be responsible for their old age, this social expectation creates a norm of behavior. Western residents perceive normative beliefs formed by family and community expectations of their children to take responsibility for their old age, and because of deep traditional beliefs, residents tend to comply with this expectation, creating a motivation for compliance. This norm reduces the willingness of the population to participate in pension insurance, as they believe that their children will take care of them in their old age without additional protection.

**Figure 2:**
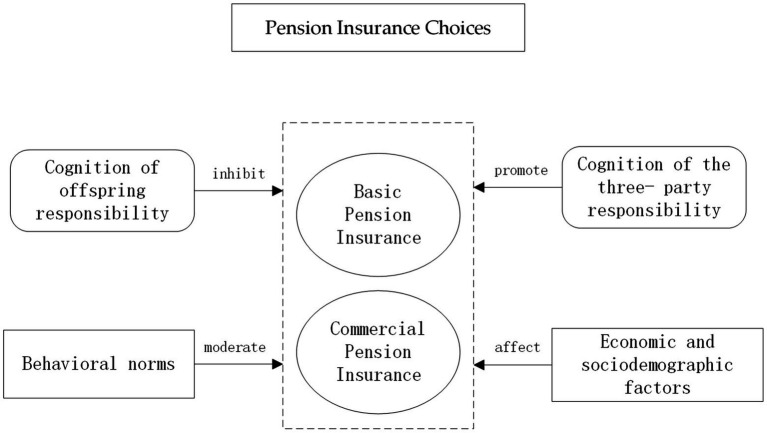
Pension insurance choices.

At the same time, the more modern and technologically advanced the information sources, the more they agree with “government responsibility.” Modern, technological information channels such as the Internet disseminate a great deal of information about the responsibility that governments should assume for old age. People perceive, through these channels, the normative belief that society expects the government to be responsible for old age and tend to rely on the government, reducing the incentive for individuals to participate in pension insurance (Figure 2).

### 5.5 Suggestions

Based on the analysis of the impact of the cognition of pension responsibility on the choice of pension insurance in this paper, we propose the following recommendations from the aspects of education, system improvement, and policy support:

First, education on the cognition of pension responsibility should be strengthened, and the notion of “three-party responsibility” should be publicized through the media, community activities, and other channels. Emphasis should be placed on the shared responsibility of the individual, the family, and society and on reducing the over-reliance on “offspring responsibility.” Courses should be conducted on pension planning in schools and communities to help the public understand the importance of pension insurance and raise their awareness of personal retirement.

Second, to support families in their old age, financial subsidies or tax incentives can be provided to children who take on the responsibility for old age, and community-based old-age services can be developed. Services such as daycare and health management should be provided to relieve family stress. At the same time, legislation should be adopted to clarify the responsibilities of the government, enterprises, and individuals in old-age protection and to ensure that all parties participate in the process.

Most importantly, the basic pension insurance system should be optimized, pensions should be adjusted in a timely manner in accordance with economic development and price levels to ensure basic living needs, and more flexibly employed persons and rural residents should be included in the scope of protection to raise the participation rate. The development of commercial pension insurance should be encouraged, participation by individuals and enterprises through tax incentives should be incentivized, and the development of diversified products by insurance companies should be promoted to meet the needs of different groups. At the same time, the construction of a multi-level pension insurance system should be promoted, encouraging enterprises through policy incentives to establish annuity plans as a supplement to basic pension insurance and advocating that individuals prepare for their old age through savings and investment, thereby reducing the burden on society and families.

### 5.6 Strengths and limitations

The strengths of this study lie not only in its utilization of the most recent pertinent data to ensure that the results closely align with current reality but also in its analysis of residents’ choice of pension insurance from the new perspective of the cognition of responsibility for pension. It also delved into the extent to which the cognition of responsibility affects people’s behavioral choices regarding pension insurance, rather than limiting the analysis to the cognition of pension responsibility itself.

However, it is important to acknowledge certain limitations of this study. First, the data employed herein originated from the Renmin University of China’s 2021 Chinese General Social Survey (CGSS) and are, therefore, secondary data. Second, the sample size utilized in this study was smaller compared to previous surveys conducted by the program, potentially elevating the influence of individual samples and, to some degree, impacting the precision of the study. The continuation of future research still needs to be supported by primary data to avoid over-reliance on secondary data.

## 6 Conclusion

Currently, the traditional notion of “offspring responsibility” for pension insurance has a significant negative impact on participation in basic and commercial pension insurance, especially in rural areas, where this notion is still deeply entrenched. This perception reduces residents’ willingness to participate in insurance schemes, weakens their awareness of risk, and affects the sustainability of contributions, thus hindering the construction of a multi-level pension security system. In contrast, the perception of “three-party responsibility” for pension insurance is more in line with the direction of development of modern social security systems, significantly increasing the willingness of residents to participate in basic pension insurance and helping to promote the construction of a multi-level security system. However, the difference between “self-responsibility” and “three-party responsibility” in terms of participation in basic pension insurance is relatively small, mainly because of the compulsory and universal nature of basic pension insurance. In the future, with changes in the concept of old age and improvement of the social security system, the influence of different cognitions of pension responsibility on participation behavior may be more obvious in other areas of old-age protection.”

## Data Availability

The original contributions presented in the study are publicly available. This data can be found at: http://cgss.ruc.edu.cn/English/Home.htm.

## References

[1] National Bureau of Statistics. Data from the Seventh Population Census [EB/OL]. Available online at: http://www.stats.gov.cn/P020230301403217959330.pdf.

[2] Wenkai S Keyong D. Research on the reform of China’s multi-pillar pension system. Macroeconomics. (2022) 11:93–103. doi: 10.16304/j.cnki.11-3952/f.2022.11.006

[3] Yang Y. Multi-Level and Multi-Pillar Pension Insurance System: Theory, Ideas and Trends[J]. Frontiers, (2022) 74–82. doi: 10.16619/j.cnki.rmltxsqy.2022.23.009

[4] Zhang B. Who will provide for the elderly in China?——is based on the analysis of Chinese cognition of pension responsibility and its influencing factors. J Huazhong Agric Univ. (2018) 4:99-109+170-171. doi: 10.13300/j.cnki.hnwkxb.2018.04.012

[5] Xu X Li P Ampon-Wireko S. The willingness and influencing factors to choose institutional elder care among rural elderly: an empirical analysis based on the survey data of Shandong Province. BMC Geriatr. (2024) 24:17. doi: 10.1186/s12877-023-04615-5 38177989 PMC10768132

[6] Bateman H Eckert C Iskhakov F Louviere J Satchell S Thorp S. Individual capability and effort in retirement benefit choice. J Risk Insurance. (2018) 85:483–512. doi: 10.1111/jori.12162

[7] Rubinstein-Levi R Kedar-Levy H. The effect of attitudes regarding retirement on pension savings. Rev Econ Fin. (2019) 15:1–13.

[8] Dragos SL Dragos CM Muresan GM. From intention to decision in purchasing life insurance and private pensions: different effects of knowledge and behavioral factors. J Behav Exp Econ. (2020) 87:101555. doi: 10.1016/i.socec.2020.101555

[9] Foster L. Understanding millennial women's attitudes towards the state pension in the United Kingdom. Age Soc. (2023) 44:2633–56. doi: 10.1017/S0144686X23000041

[10] Xinxin W Qingqing Z. Family parenting burden, willingness and behavior of pension insurance: an analysis based on the "Sandwich class" of floating population. Insurance Stud. (2021) 8:83–96. doi: 10.13497/j.cnki.is.2021.08.006

[11] Liu Y Luo X Xu H. Economic autonomy as a determinant of physical activity behavior in Chinese older adults. Front Public Health. (2025) 12:1466710. doi: 10.3389/fpubh.2024.1466710, PMID: 39980618 PMC11841652

[12] Zhang T Lu B Wang X. Urban-Rural Disparity in Cognitive Performance Among Older Chinese Adults: Explaining the Changes From 2008 to 2018. Front Public Health. (2022) 10:843608. doi: 10.3389/fpubh.2022.843608 35400051 PMC8984104

[13] Nick S Saurabh P. Regulation without reason: the deleterious effects of government regulation on private pension provision. Econ Aff. (2012) 3:50–7. doi: 10.1111/j.1468-0270.2012.02174.x

[14] Gelissen J. Old-age pensions: individual or collective responsibility? An investigation of public opinion across European welfare states. Eur Soc. (2001) 3:495–523. doi: 10.1080/14616690120112235

[15] Casey BH Dostal JM. Voluntary pension saving for old age: are the objectives of self-responsibility and security compatible? Soc Policy Adm. (2013) 47:287–309. doi: 10.1111/j.1467-9515.2012.00853.x

[16] Riekhoff AJ. Pension reforms, the generational welfare contract and preferences for pro-old welfare policies in Europe. Soc Policy Adm. (2021) 55:501–18. doi: 10.1111/spol.12678

[17] Wang Y Zhang R Peng S. Cognitive differences and influencing factors of Chinese People’s old-age care responsibility against the ageing background. Healthcare. (2021) 9:72. doi: 10.3390/healthcare9010072, PMID: 33466631 PMC7828671

[18] Cui Y He Y Xu X Zhou L Nutakor JA Zhao L. Cultural capital, the digital divide, and the health of older adults: a moderated mediation effect test. BMC Public Health. (2024) 24:302. doi: 10.1186/s12889-024-17831-4 38273305 PMC10811880

[19] Fishbein M Ajzen I. Belief, attitude, intention, and behavior: an introduction to theory and research. Addison-Wesley (1977).

[20] Ketkaew C Sukitprapanon C Naruetharadhol P. Association between retirement behavior and financial goals: a comparison between urban and rural citizens in China. Cogent Bus Manag. (2020) 7:1739495. doi: 10.1080/23311975.2020.1739495

[21] Xu S Ali ST Yang Z Li Y. Effect of household’s financial literacy on pension decision making: evidence from China’s new rural pension program. Kybernetes. (2023) 52:4611–44. doi: 10.1108/K-03-2022-0321

[22] Jensen B. ‘Perceived social citizenship’: a comparative study between two different hukous. Citizsh Stud. (2019) 23:172–88. doi: 10.1080/13621025.2019.1584157, PMID: 40101104

[23] Kwak C Clayton-Matthews A. Multinomial logistic regression. Nurs Res. (2002) 51:404–10. doi: 10.1097/00006199-200211000-00009 12464761

[24] Liao W Liu M. Family Environment,Generation Difference and Residents’ Cognition of Pension Responsibility: An Empirical Study Based on the Data of CGSS2015 One-child Family[J]. Northwest Population Journal, (2020) 41:67–78+89. doi: 10.15884/j.cnki.issn.1007-0672.2020.02.006

[25] Xiao H Song Y. Relationship between cultural capital, physical exercise and subjective health of urban residents in China: empirical analysis based on CGSS 2017 data. J Xi'an Phys Educ Univ. (2022) 39:570–80. doi: 10.16063/j.cnki.issn1001-747x.2022.05.007

[26] Li H Chen L Zhang Z. A study on the utilization rate and influencing factors of small agricultural machinery: evidence from 10 hilly and mountainous provinces in China. Agriculture. (2023) 13:51. doi: 10.3390/agriculture13010051

[27] Woo-Yung K. Subways and the labor force participation of Females: The case of Daejeon, Korea. Research in Transportation Economics, (2019) 75:69–82. doi: 10.1016/j.retrec.2019.04.001

[28] Yan N Ren L Xiang Y. Study on the behavior of participating in basic endowment Insurance for Young net Workers in new form of employment based on TPB framework: Micro-empirical study based on questionnaire survey. J China Univ Geosci. (2024) 24:143–56. doi: 10.16493/j.cnki.42-1627/c.20231130.001

[29] Liu X Fan Y Li C. Research on the influence of new family concept on commercial pension insurance participation: empirical evidence based on CFPS2018 survey data. Northwest Pop J. (2022) 43:118–26. doi: 10.15884/j.cnki.issn.1007-0672.2022.04.010

